# Cardiac disease and arrhythmogenesis: Mechanistic insights from mouse models

**DOI:** 10.1016/j.ijcha.2016.05.005

**Published:** 2016-05-14

**Authors:** Lois Choy, Jie Ming Yeo, Vivian Tse, Shing Po Chan, Gary Tse

**Affiliations:** a School of Biomedical Sciences, Li Ka Shing Faculty of Medicine, University of Hong Kong, Hong Kong; b School of Medicine, Imperial College London, SW7 2AZ, UK; c Department of Physiology, McGill University, Canada

**Keywords:** Mouse model, Cardiac arrhythmia, Cardiomyopathy, Ion channelopathy, Conduction, Repolarization

## Abstract

The mouse is the second mammalian species, after the human, in which substantial amount of the genomic information has been analyzed. With advances in transgenic technology, mutagenesis is now much easier to carry out in mice. Consequently, an increasing number of transgenic mouse systems have been generated for the study of cardiac arrhythmias in ion channelopathies and cardiomyopathies. Mouse hearts are also amenable to physical manipulation such as coronary artery ligation and transverse aortic constriction to induce heart failure, radiofrequency ablation of the AV node to model complete AV block and even implantation of a miniature pacemaker to induce cardiac dyssynchrony. Last but not least, pharmacological models, despite being simplistic, have enabled us to understand the physiological mechanisms of arrhythmias and evaluate the anti-arrhythmic properties of experimental agents, such as gap junction modulators, that may be exert therapeutic effects in other cardiac diseases. In this article, we examine these in turn, demonstrating that primary inherited arrhythmic syndromes are now recognized to be more complex than abnormality in a particular ion channel, involving alterations in gene expression and structural remodelling. Conversely, in cardiomyopathies and heart failure, mutations in ion channels and proteins have been identified as underlying causes, and electrophysiological remodelling are recognized pathological features. Transgenic techniques causing mutagenesis in mice are extremely powerful in dissecting the relative contributions of different genes play in producing disease phenotypes. Mouse models can serve as useful systems in which to explore how protein defects contribute to arrhythmias and direct future therapy.

## 1 Introduction

The mouse is the second mammalian species, after the humans [12], in which substantial amount of the genomic information has been analyzed [13]. With advances in transgenic technology [14], mutagenesis is now much easier to carry out in mice [15]. Consequently, an increasing number of transgenic mouse systems have been generated for the study of cardiac arrhythmias [16], [17]. These models can be loosely divided into ion channelopathies with minimal structural abnormalities, and those of structural heart disease. The former group includes catecholaminergic polymorphic ventricular tachycardia (CPVT) [18], the long [19] and short QT syndromes (LQTS and SQTS), and Brugada syndrome (BrS) [20]. The latter group includes several types of cardiomyopathies, such as arrhythmogenic right ventricular dysplasia (ARVD) [21], dilated cardiomyopathy (DCM) [22] and hypertrophic cardiomyopathy (HCM) [23]. However, now it is much clearer that structural alterations are found in ion channelopathies; for example, myocardial fibrosis is observed in BrS [24], [25], [26], DCM and non-compaction cardiomyopathy features are found in cardiac ryanodine receptor 2 mutation that is classically observed in CPVT [27]. Conversely, cardiomyopathy has been associated with ion channel mutations, as exemplified by sodium channel mutation in DCM [28]. Thus, these categories inevitably contain some overlap. As previously suggested, a better classification of cardiomyopathy includes additional subtypes affecting the cytoskeleton, desmosome, sarcomere and ion channels [29]. Some authors have asserted that this classification is too complex for clinical use, proposing instead a “MOGES” classification based on “morphofunctional phenotype (M), organ(s) involvement (O), genetic inheritance pattern (G), etiological annotation (E) including genetic defect or underlying disease/substrate, and the functional status (S) of the disease” [30]. Atrial fibrillation is a particularly complex disease, involving an interplay between electrical and structural remodelling, autonomic imbalance, alterations in calcium handling and genetic factors [31]. Mouse studies have illustrated the importance of abnormal metabolism in the initiation of paroxysmal atrial fibrillation and its progression to persistent and permanent forms [32], and shed light on the electrophysiological abnormalities predisposing to arrhythmias [33], but will not be discussed further in this review.

**Fig. 1:**
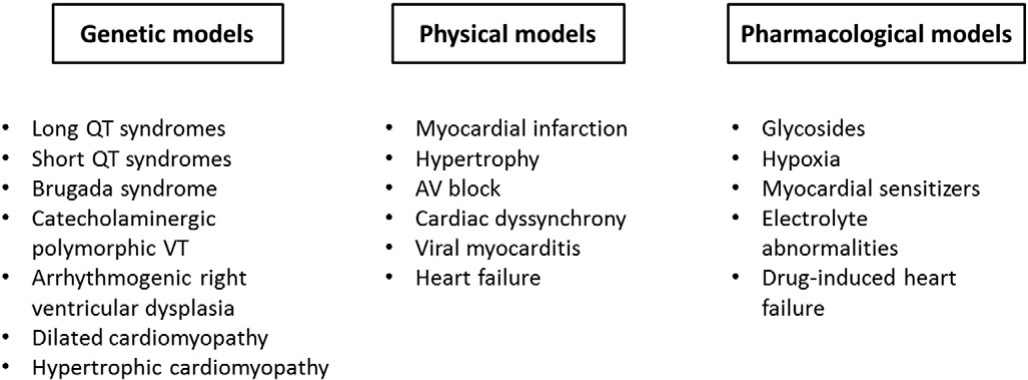
Genetic, physical and pharmacological models in mouse hearts.

Non-genetic mouse models have also been used for the study of human cardiovascular conditions and associated arrhythmic properties [34]. Physical models include myocardial infarction produced by coronary artery ligation [35], hypertrophy and heart failure by transverse aortic constriction [36], complete AV block model by radiofrequency ablation of the AV node [37] and cardiac dyssynchrony model by implantation of a miniature pacemaker tailored to mouse hearts [38]. In contrast, pharmacological models include the use of cardiac glycosides [39], hypoxia [40], myocardial sensitizers [41] such as chloroform [42], [43] and alterations in electrolyte concentrations, for example, potassium [44]. Cardiac toxins such as ethanol [45] and doxorubicin [46] have been used for modelling heart failure [47]. The different types of models are summarized in Fig. 1. In the next section, the advantages and disadvantages of mouse models, and comparisons between human cardiac physiology and mouse cardiac physiology will be discussed. The current understanding of each pathology, focusing on how mouse models have aided understanding, will then be reviewed in turn.

## 2 Advantages and disadvantages of mouse models

Several reasons justify the use of mice to study of human arrhythmia conditions. Firstly, 99% of mouse genes have a homologue in the human genome [13]. Secondly, both species have a similar set of ion channel genes [48]. Thirdly, the vast majority of these ion channel genes have nearly identical sequence homology in both species [49]. Fourthly, these genes have similar expression patterns, and their protein products show similar structural, electrophysiological [50] and pharmacological properties [48]. Finally, the same mutation in ion channel genes can often produce similar phenotypes in both species. For example, genetically engineered mice with altered potassium channel expression show prolonged ventricular action potential durations (APDs), prolonged electrocardiographic QT intervals and increased arrhythmogenicity, closely recapitulating the findings in the corresponding human conditions [51]. There are also advantages of using mice. The first relates to their vulnerability to arrhythmias. The mouse heart is electrically more stable than the human heart because of its small size, and spontaneous ventricular arrhythmias are therefore less likely to occur [49]. This means a smaller number of mice are lost due to unwanted lethal arrhythmias, potentially saving costs. The second relates to the relative ease of defibrillation. Arrhythmias are easier to reverse in mice than in larger species, making them invaluable for the evaluation of the effectiveness of anti-arrhythmic drugs. However, caution must be taken because efficacy could be overestimated.

## 3 Comparisons between human and mouse cardiac electrophysiology

Mouse hearts are similar to human hearts in many respects, making them invaluable as model systems for the study of human arrhythmic syndromes. Firstly, the SA and AV nodes as well as the His-Purkinje system are structurally similar [52]. Secondly, the same patterns of depolarization and repolarization are observed in both species, with depolarization spreading from endocardium to epicardium and from apex to base, and repolarization from epicardium to endocardium and from base to apex [53]. Thirdly, the transmural conduction velocities (CVs) are nearly identical in mouse and human hearts [53]. Fourthly, apex-base and endocardium–epicardium repolarization gradients are present in both species [54]. Finally, the upstroke of the action potential in both mice and humans is attributed to *I*_Na_, making mouse hearts especially suitable for studying changes in CV [55]. Readers who are interested in the electrophysiological mechanisms of arrhythmogenesis are directed to these review articles [56], [57], [58].

However, it must also be recognized that mice do show some important differences in their cardiac electrophysiology [51], [53], [59]. Firstly, the basal heart rate in the mouse is around 600 bpm, which is ten times greater than that observed in the human [60]. Secondly, the morphology of the mouse and human ventricular action potentials is different, with the mouse ventricular action potential having a shorter duration and lacking a plateau phase [61]. This has been attributed to different expression levels of repolarizing potassium channels. Thus, *I*_to_ is the major repolarization current with *I*_Kr_ and *I*_Ks_ having a diminished role in mice [62], whereas *I*_Kr_ and *I*_Ks_ are the major repolarization currents in humans [63]. Additional differences between mouse and human electrophysiology lead to difficulties in extrapolating data obtained from mice to humans and interpreting the mouse electrocardiogram [64]. Other species such as guinea pigs [65], [66], [67], [68], [69], [70], [71] and rabbits [72], [73] may be better models for studying cardiac repolarization, as their ion currents are similar to those found used by human hearts.

**Fig. 2:**
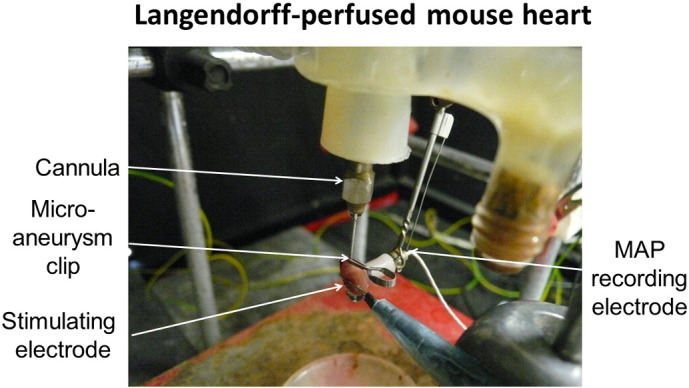
Experimental setup for Langendorff perfusion, which monophasic action potential (MAP) recordings during simultaneous pacing.

The critical mass hypothesis posited that heart size must be sufficiently large to support fibrillation [74]. Because the wavelength of the excitation, given by ERP x CV, must be smaller than the available path length to allow re-entry [75], mouse hearts were originally thought to be too small to sustain re-entrant pathways. However, reconstruction of activation pattern [64] and mapping studies [19], [76], [77] have both shown that re-entry can take place. Bearing these limitations in mind, mouse models have provided significant advances in our understanding of cardiac electrophysiology. It is made possible by monophasic action potential (MAP) and bipolar electrogram (BEG) techniques to examine local activation and repolarization patterns [78], [79], [80], [81]. Fig. 2 shows an experimental setup for recording left ventricular epicardial MAPs from isolated, Langendorff-perfused mouse hearts during right ventricular pacing. The study of congenital ion channelopathies has provided much insights into the general mechanisms by which disturbances in action potential conduction and repolarization generate arrhythmias, whereas that of heart failure and atrial fibrillation have identified pathological processes underlying disease progression with time and age [82]. These conditions will be discussed in turn.

## 4 Catecholaminergic polymorphic ventricular tachycardia (CPVT)

**Fig. 3:**
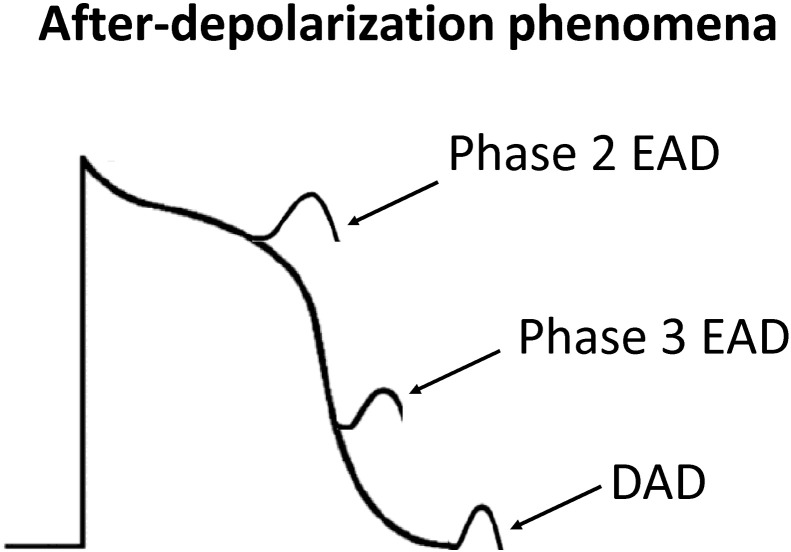
Afterdepolarization phenomena: early afterdepolarization (EAD) occurs early (phase 2) or late (phase 3), and delayed afterdepolarization (DAD) occurs during phase 4 of the action potential. When sufficiently large, these can result in triggered activity.

Reproduced from Tse (2015) [58] with permission.

Catecholaminergic polymorphic ventricular tachycardia (CPVT) is an inherited, cardiac ion channelopathy characterized by adrenaline-driven ventricular arrhythmias [83]. Clinically it manifests frequently as bidirectional ventricular tachycardia (VT), which is inducible by exercise stress testing [84], [85]. Other presentations include polymorphic VT and survivors of cardiac arrest [85]. An autosomal dominant form, CPVT1, was first found to be associated with mutations in the gene encoding for the cardiac ryanodine receptor 2 (RyR2), which releases calcium from the sarcoplasmic reticulum [86]. To date, some 150 mutations in RyR2 have been implicated in CPVT [85]. Recently, a large genomic deletion of human cardiac RYR2 gene, resulting from in-frame deletions of exon-3 (Ex3-del), has been detected in several, unrelated families [27], [87], [88], [89]. The proposed mechanism of arrhythmogenesis in CPVT has traditionally been delayed afterdepolarization (DAD) phenomena leading to triggered activity (Fig. 3) [90]. This has been attributed to a leaky RyR2 leading to calcium release [91]. However, this mechanistic scheme is an inadequate for arrhythmias due to loss-of-function RyR2 mutations, for which murine studies have provided much insight into the underlying physiological mechanisms [92]. CPVT2, an autosomal recessive form, is associated with missense mutations of the gene encoding for calsequestrin 2 (CASQ2), a calcium-binding protein of the sarcoplasmic reticulum [93], [94]. Affected individuals were homozygous for the D307H missense mutation [93], [94]. Interestingly, three other missense mutations in CASQ2 have been described in particular severe forms of CPVT, with possible autosomal dominant or oligogenic inheritance [95].

In mice, several gain-of-function mutations in RyR2 have been generated, shedding light onto the molecular mechanisms of arrhythmogenesis. Heterozygous knock-in mice carrying the RyR2 (R4496C) mutation shows increased calcium sensitivity of RyR2, leading to diastolic calcium release [96]. Interestingly, experiments in single cells from this system showed that increasing the sarcoplasmic reticulum calcium overload alone is sufficient to induce DAD activity through activation of the sodium–calcium exchanger (NCX) [97]. This took place even in an absence of beta adrenergic stimulation [97]. In homozygous RyR2-P2328S mice, abnormal calcium handling has been associated with reduced CVs, predisposing the hearts to spontaneous ventricular arrhythmias [98]. Studies using isolated myocytes showed that the abnormal functions of RyR2 have been rescued by increasing the affinity of calmodulin to RyR2, which reduced the frequency of DADs and triggered activity [99]. In contrast, mouse models for loss-of-function RyR2 mutation can be exemplified by RyR2-A4860G heterozygotes [92]. Isolated myocytes from these mice showed a reduced amplitude of calcium release during systole, leading to calcium overload of the sarcoplasmic reticulum [92]. This in turn caused random bursts of prolonged calcium release, activation of NCX with consequent early after-depolarization (EAD) phenomena. Furthermore, heterozygous Ex3-del mice (Ex3-del^+/−^) modelling the corresponding human condition failed to show susceptibility to CPVT [100]. Nevertheless, cardiac specific, conditional knockout of the wild-type RyR2 allele in Ex3-del^+/−^ mice led to bradycardia and sudden death [100], closely recapitulating the findings observed in humans. This would suggest there are variations in the arrhythmic phenotype. Finally, genetically modified mice bearing CASQ2 mutation showed decreased refractoriness of the RyR leading to spontaneous diastolic calcium release, and hence the development of DADs [101].

**Fig. 4:**
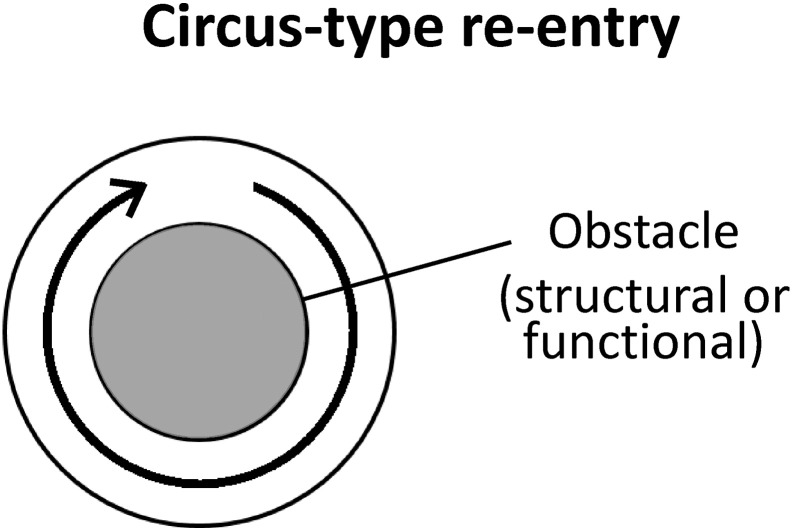
Circus-type reentry requires a structural or functional obstacle (gray center) around which an action potential can circulate.

Reproduced from Tse (2015) [58] with permission.

In summary, the above mouse models implicate abnormal calcium handling as a predominant mechanism underlying arrhythmogenesis in CPVT. This in turn results in an inward current by activation of the NCX, and in turn to EADs or DADs, which can induce triggered activity. Abnormal calcium handling may also lead to reduced CV of action potential propagation, additionally serving as a substrate for circus re-entry (Fig. 4).

## 5 Long QT syndromes (LQTS)

**Fig. 5:**
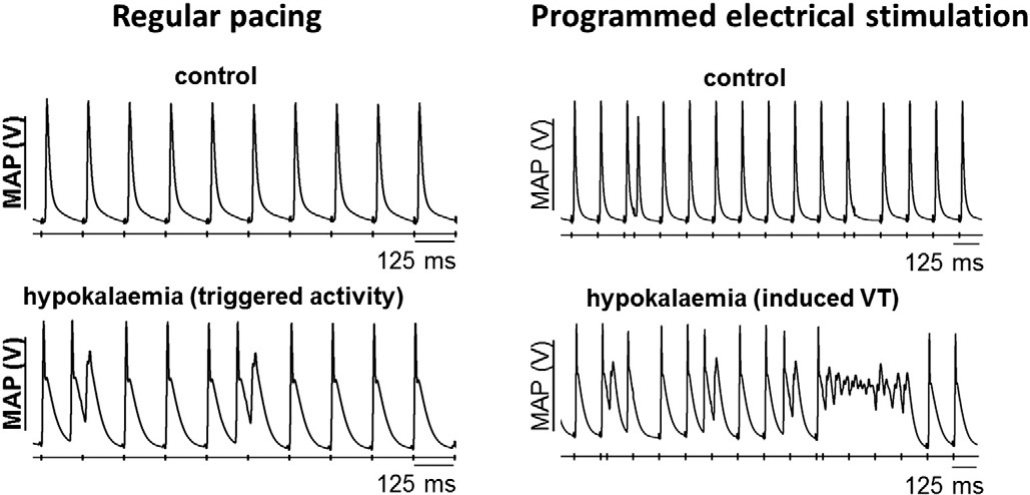
Hypokalaemia prolongs APDs, which predisposes to triggered activity (left). This AP prolongation and reduced refractoriness together form a re-entrant substrate. The use of programmed electrical stimulation can reliably provoke ventricular arrhythmias (right).

**Fig. 6:**
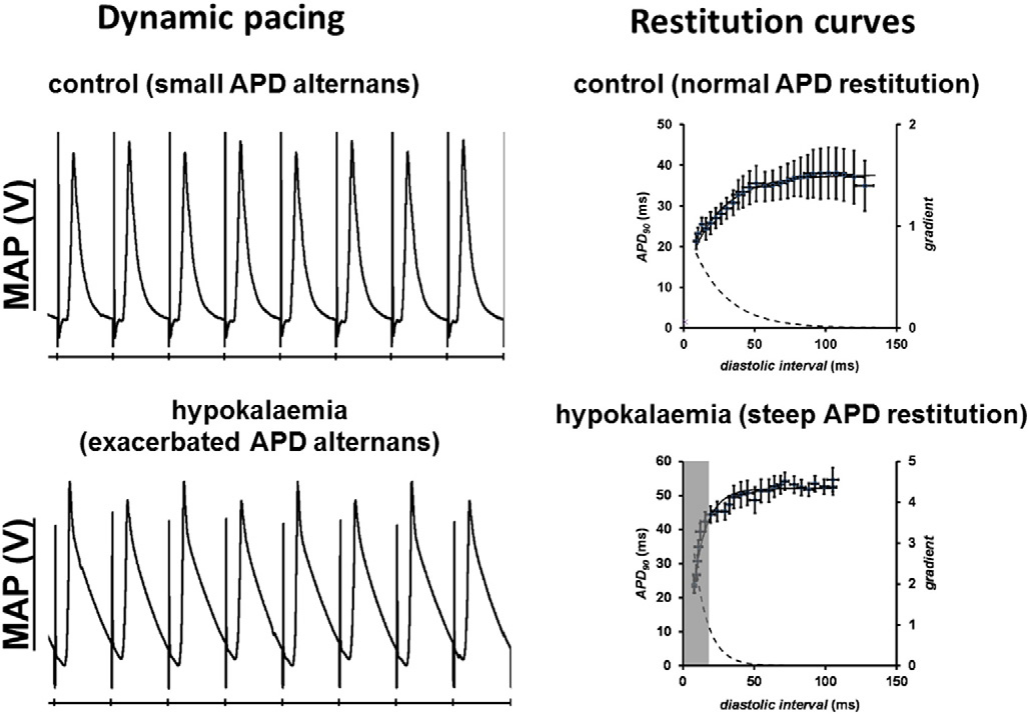
Hypokalaemia exacerbates APD alternans at fast heart rates (left) due to steep APD restitution (right).

Long QT syndromes (LQTS) refers to a group of disorders characterized by a prolonged QT interval of ≥ 450 ms on the electrocardiogram (ECG), with congenital or acquired causes. The underlying electrophysiology involves either reduced repolarizing currents or increased depolarizing currents. Congenital LQTS has two hereditary variants: Romano–Ward syndrome is inherited in an autosomal dominant manner, and Jervell and Lange–Nielsen (JLN) syndrome shows autosomal recessive inheritance associated with congenital deafness [102]. Thirteen LQTS subtypes have been identified thus far. LQTS 1 (KCNQ1), 2 (KCNH2), 5 (KCNE1), 6 (KCNE2), 7 (KCNJ2) and 13 (KCNJ5) are caused by loss-of-function mutations in genes encoding for the different potassium channels. LQTS 3 (SCN5A) and 10 (SCN4B) are caused by gain-of-function mutations in genes for sodium channel subunits. LQTS 8 (CACNA1C), called Timothy syndrome, is caused by gain-of-function mutations in the L-type calcium channel. LQT 4 (ANKB), 9 (CAV3), 11 (AKAP9) and 12 (SNTA1) are caused by mutations in other supporting proteins. Acquired causes of LQTS are much more common than congenital syndromes. This is exemplified by hypokalaemia, which is the most common electrolyte abnormality encountered in clinical practice. A mouse pharmacological model of experimental hypokalaemia has demonstrated the following electrophysiological mechanisms: APDs are prolonged, predisposing to EADs and therefore triggered activity (Fig. 5) [44], [103]. This prolongation preferentially occurs at the epicardium compared to the endocardium, leading to a reversal of the transmural repolarization gradient [44]. This in combination with reduced refractory periods [104], produced a favourable substrate for re-entrant arrhythmias that can readily be induced by programmed electrical stimulation. Moreover, altered cardiac dynamics, particularly the onset of APD alternans, attributed to increased steepness of action potential restitution, also contribute to re-entrant substrates (Fig. 6) [57], [105]. It was shown that loss of gap junction function paradoxically exerted anti-arrhythmic effects, by reversing changes in refractory periods despite leaving APD and CV restitution unaffected [103], [105]. Modulation of gap junctions may therefore be a viable therapeutic strategy for anti-arrhythmic therapy [106], [107], [108], [109].

Traces reproduced from Tse et al. (2016) [103] with permission.

Traces reproduced from Tse et al. (2016) [105] with permission.

## 6 Short QT syndromes (SQTS)

Short QT syndrome is a group of heterogeneous conditions characterized by an abbreviated QT interval (QTc < 350ms). It causes an increased risk of atrial and ventricular arrhythmias, in particular ventricular fibrillation, thereby predisposing to sudden cardiac death. The shortening of QT interval could be contributed by an increased activity of repolarizing currents, or reduced activity of depolarizing currents. Six genetic subtypes of SQTS have been identified thus far. Gain-of-function mutations in the potassium channel genes, KCNH2, KCNQ1 [110], [111] and KCNJ2 [112] are found in SQT1, 2 and 3. By contrast, loss-of-function mutations in L-type calcium channel subunits, CACNA1C, CACNB2 and CACNA2D1, are found in SQT4, 5 and 6, respectively [113]. Interestingly, altered cardiac metabolism can also lead to a SQT phenotype, as exemplified by mice treated with mildronate, which resulted in low carnitine and shortened QT intervals [114]. The common electrophysiological abnormality involves shortened APD, which reduces the excitation wavelength and thereby predisposing to circus-type re-entry [58]. Unlike the long QT syndromes, abnormal APD restitution leading to alternans formation, which involves APD prolongation, is unlikely to be a contributing factor in arrhythmogenesis [57].

## 7 Brugada syndrome (BrS)

In Brugada syndrome, there is loss-of-function mutations in the *SCN5A* gene, predisposing affected individuals to ventricular arrhythmias and sudden death [115]. There has been considerable debate on the mechanisms of arrhythmogenesis in BrS. The leading theories are the depolarization and repolarization hypotheses [116]. The depolarization hypothesis proposes that mild structural abnormalities leading to conduction disturbances, which would predispose to circus-type re-entry. By contrast, the repolarization hypothesis depends on transmural dispersion of repolarization between endocardium and epicardium in the right ventricular outflow tract (RVOT). Experiments performed on mouse models have shed some light on the underlying mechanisms. Thus, heterozygous targeted disruption of Scn5a (Scn5a^+/−^) mice showed conduction abnormalities associated with fibrosis in the right ventricle [117]. Adult mice heterozygous for a mutation associated with BrS (Scn5a^1798insD/+^) showed reduced CVs in the RVOT [118], and those possessing the SCN5a^G1408R^ mutation showed slowed conduction, shortened APDs despite prolonged refractory periods associated with mild interstitial fibrosis [119]. This would suggest contributions from both depolarization and repolarization abnormalities to the arrhythmic phenotype. Reduced sodium current can also arise from dysfunction of other proteins, such as the desmosomal component plakophilin-2. Patients with missense mutations of this protein show a reduced number of sodium channels at the intercalated disc associated with a Brugada phenotype. Interestingly, mouse hearts with plakophilin-2missense mutations show a phenotype that is more consistent with ARVD, as described below [120]. This in turn suggests that although both ARVD and BrS are primarily right ventricular diseases, there is a phenotypical spectrum with decreasing severity of structural abnormalities away from ARVD towards BrS.

## 8 Arrhythmogenic right ventricular dysplasia (ARVD)

Arrhythmogenic right ventricular dysplasia (ARVD) is a primary cardiomyopathy characterized by fibro-fatty replacement of the right ventricular myocardium, predisposing affected individuals, particularly in young adults and athletes, to ventricular arrhythmias and sudden cardiac death [121]. The disease progresses to ventricular wall thinning and development of aneurysms [122]. Approximately 50% of the patients have defects in their desmosomal components [123]. Desmosomes consist of cadherins, armadillo proteins (which include plakoglobin and plakophilin 2, PKP2) and desmoplakin, which links this complex to desmin in the intermediate filament [124]. The culprits for the remaining 50% are elusive, but mouse models have provided much insight into the different proteins affected. Inhibitor of apoptosis-stimulating protein of p53 (iASPP) is a protein expressed in intercalated discs, interacting with desmoplakin [125] and its deficiency was shown to induce features of ARVD in mice [125]. Rho-kinase inhibition in the developing mouse heart (SM22α-restricted) show similar physical findings [126]. Although traditionally recognized as a predominant right ventricular disease, left-dominant and biventricular involvement has been described [127]. Mouse models have advanced our understanding, in particular implicating desmoplakin deficiency as a cause in left-ventricular and biventricular dominant forms [128], supporting similar findings observed in humans [129].

In humans, recent studies have implicated exercise and endurance training as precipitants or triggers of arrhythmias [130], [131]. Heterozygous plakoglobin-deficient mice showed accelerated development of structural abnormalities and arrhythmias following endurance training [132]. Moreover, missense mutation of the plakophilin-2 gene (PKP2) demonstrated right ventricular systolic dysfunction and regional wall motional abnormalities on cardiac magnetic resonance imaging in exercise trained but not sedentary mice [120]. This would suggest an unmasking of an otherwise quiescent arrhythmic phenotype inducible by exercise. Together, both clinical and mouse studies provide evidence that exercise restriction prevent the occurrence of ventricular arrhythmias in ARVD patients.

## 9 Dilated cardiomyopathy (DCM)

Dilated cardiomyopathy is a type of primary cardiac muscle disorder characterized by increased ventricular mass and dilatation associated with impaired mechanical function [133]. It predisposes affected individuals to ventricular arrhythmias and sudden death. Only 30% of DCM cases are familial [133]. To date, two X-linked and 31 autosomal genetic mutations have been identified as the causes of DCM [134], [135]. Often these are proteins that are responsible for maintaining structural integrity of the cardiomyocyte, such as myosin heavy chain (MHC), myosin-binding protein C, alpha tropomyosin, and titin, which is the largest protein identified thus far. Other causes include environmental factors, such as viral infections by enteroviruses and adenovirus, which result in myocarditis and subsequently DCM [136]. Mouse models have been useful in elucidating the ion channel abnormalities and electrophysiological mechanisms underlying arrhythmogenesis in this condition. For example, homozygous mutant mice expressing a truncated form of myosin-binding protein C (MyBP-C^t/t^) develop severe dilated cardiomyopathy, exhibited extensive cardiac fibrosis associated with inducible ventricular tachycardia (AT) despite surprisingly normal conduction and refractoriness [137].

Interestingly, DCM has been associated with reduced SCN5a expression [138]. Thus, in transgenic mice that ectopically express the transcriptional repressor Snail, there was a severe DCM phenotype together with reduced sodium current and conduction slowing without changes in intercellular coupling. The mechanism of arrhythmia here may be circus-type re-entry, which requires conduction slowing. Moreover, mice with the D1275N mutation in SCN5a showed features of DCM, reduced CVs, heart block and ventricular arrhythmias [139]. Mice with a deletion mutation ΔK210 in cardiac troponin T had DCM, APD prolongation secondary to reductions in transient outward (I_to_) and ultrarapid delayed rectifier K + (I_Kur_) currents [140]. This predisposed them to both early and delayed afterdepolarization phenomena and therefore triggered activity. A significant finding is that potassium channel downregulation was observed even when there was no evidence of heart failure [140]. This thereby implicates ion channel remodelling as an important mechanism for arrhythmogenesis in DCM, and already takes place before additional electrophysiological abnormalities associated with heart failure, such as NCX upregulation, become apparent. Together, these findings in mice are consistent with those in humans, where SCN5a mutations have also been implicated in the pathogenesis of DCM [141].

In other mouse model, where mice overexpressing dominant-negative neuron-restrictive silencer factor (NRSF), a transcriptional regulator of myosin, developed a DCM phenotype [142]. These mice showed increased expression of I_f_ and I_Ca,T_, which are usually expressed in pacemaker cells, suggesting that increased automaticity may also be an underlying arrhythmogenic mechanism in DCM. In cardiomyocytes, the cytoskeleton is in close association with the nuclear envelope, aided by inner nuclear membrane proteins called lamins. Heterozygous lamin knockout (Lmna^+/−^) mice showed DCM phenotype associated with increased susceptibility to AV nodal disease as well as ventricular tachycardia [143].

## 10 Hypertrophic cardiomyopathy (HCM)

Hypertrophic cardiomyopathy is the commonest inherited cardiac disease characterized by a non-dilated, hypertrophied left ventricle without other causes of hypertrophy, such as storage, infiltrative disease or pressure overload, being observed [144]. Its genetics are less heterogeneous than DCM, as mutations in the genes that encode for the myosin heavy chain, myosin binding protein C and troponin T are responsible for about 75% of all inherited HCMs [145]. Heterozygous MyBP-C^t/+^ mice showed a mild HCM and inducible ventricular tachycardia after pharmacological stress with isoproterenol [137]. The duration of the VT is less than the homozygous MyBP-C^t/t^ mice showing a severe DCM phenotype described above. Like DCM, in mice bearing the MHC mutation (MHC^403/+^), it is the degree of hypertrophy, but not the extent or location of myocardial disarray and ventricular fibrosis, that correlated with increased arrhythmogenicity [146]. Altered calcium sensing of the myofilament caused by troponin T mutation resulted in HCM, shortened ventricular effective refractory periods, increased dispersion of CVs and increased APD alternans, all of which would predispose to re-entry [147]. Moreover, mice with the cardiac troponin T I79N mutation showed APD shortening and increased diastolic calcium release in the presence of stress [148]. This could potentially result in DAD-induced triggered activity, but remains to be studied in the future. Since ion channels can be abnormal in cardiomyopathies, they are potential targets for therapy. For example, inhibitor of the late sodium current reversed by mechanical and electrical dysfunction in HCM patients [149]. Mouse models are therefore an attractive system in which to examine the therapeutic effects of experimental drugs.

## 11 Heart failure

Heart failure is a significant burden on our healthcare system, and represents a common pathway of many aetiologies with an abysmal clinical outcome [150]. It is characterized by structural abnormalities of left ventricular dysfunction and dilatation, a compensatory rise in systemic vascular resistance secondary to activation of neurohumoral pathways [151], inflammation and metabolic adaptations to substrate utilization [152]. The net effect is comprised cardiac contractility and the inability to meet metabolic demands of peripheral tissues [153]. Cardiac dyssynchrony is a feature observed in heart failure, referring to the disruption in both temporal and mechanical coordination between the contractive motions of different cardiac compartments. It can be divided into atrioventricular, interventricular and intraventricular dyssynchrony.

The commonest heart failure model involves surgery intervention. Generation of these models allow the different stages of heart failure to be examined. Coronary artery ligation mimics myocardial infarction [35], [154], producing scarring of the myocardium followed by dilatation of the ventricle. Novels methods of coronary artery ligation have recently been devised to model ischaemia-reperfusion [155], [156], which would be ideal for studying arrhythmias associated with this phenomenon. In contrast, transverse aortic banding simulating pressure overload from aortic stenosis or hypertension triggers hypertrophic response to that similarly observed in humans [36], [157]. Non-invasive imaging modalities such as strain-based modelling revealed areas of dyssynchrony when there is no overt heart failure [158]. This supports the notion that regional, as opposed to global, stress is induced by increased afterload. The risk of ventricular arrhythmias is reduced when intraventricular dyssynchrony improves in humans [159]. Mouse models will prove useful to examine whether resynchronization of the failing heart can lower arrhythmic risk. Genetic models have allowed the investigation of the roles of each gene in heart failure and determines whether particular alterations of gene expression represent adaptive or maladaptive responses [160], [161]. Mutagenesis has been coupled with coronary artery ligation techniques to examine the propensity to arrhythmias. Thus, toll-like receptor 2 deficiency confers a protective effect against ventricular arrhythmias and reduces infarct size [162], whereas cardiac knockout of mitochondrial uncoupling protein 3 shows poorer ventricular function and larger infarct size compared to wild-type [163].

## 12 AV block

AV block refers to a group of disorders characterized by partial or complete interruption of impulse transmission from the atria to the ventricles. Depending on the extent of impulse interruption, it can be classified into first, second and third-degree, each with characteristic appearance on the electrocardiogram. Approximately 50% of patients with AV block have idiopathic fibrosis and sclerosis of the conduction system, 40% have ischaemic heart disease and the remaining minority of cases can be attributed to valvular disease, increased vagal tone and congenital bradycardia syndromes [164], [165]. This condition can also be caused by drugs, such as fingolimod, pro-drug of a S1P-R modulator licensed for multiple sclerosis [166]. Patients suffering from complete atrioventricular block (CAVB) have an increased risk of sudden cardiac death, potentially from QT prolongation and VT. Mouse models have provided much insight of the electrophysiological mechanisms underlying idiopathic AV block. It is now recognized that several proteins are responsible for the maintenance of AV conduction [167], such as T-type calcium channels [168], L-type calcium channels [169], HCN channels [170], and Sphingosine-1-phosphate receptor [171].

Mice with radiofrequency ablation of the AV node exhibited electrophysiological modelling preceded structural remodelling, which were associated with increased QT intervals and occurrence of polymorphic VT [172]. Thus, K_v_4.2 channels were downregulated with a consequent reduction in the transient outward potassium current. These changes were followed by biventricular hypertrophy and heart failure, as reflected in transcriptional changes such as rise in α-actin, β-MHC and B-type natriuretic peptide and fall in SERCA2 expression. Transgenic mouse models have also provided additional insight. Mice with deletion of Ca_v_3.1, which encodes for the α-subunit of the T-type calcium channel, was used in combination with radiofrequency AV node ablation [173]. These mice showed hypertrophic remodeling after AV block and were more prone to bradycardia-related ventricular arrhythmias.

Current treatment of AV block is by pacemaker implantation. With consideration of the shortcomings of electronic pacemakers, genetic manipulation of specific ion channels for biological pacemaker development has been an area of intense research. Thus, non-viral gene transfer with poloxamine nanosphere as the gene delivery system was used to induce overexpression of the pacemaker current-generating channel HCN2 and β_2_-adrenergic receptor (ADRB2), with the generation of functional biological pacemakers and improvement in life expectancy of mice with CAVB [37]. Recently, a S1P_3_ receptor antagonist (SPM-354) was found to restore sinus rhythm and reverse CAVB [174]. Stem cell therapy has also demonstrated promising results. Recently, brown adipose tissue (BAT)-derived cells were injected intramyocardially around the AV node, which full or partial recovery to sinus rhythm or second degree 2:1 block [175].

## 13 Conclusion

This article reviewed the different genetic and physical models of cardiovascular diseases, and explored how mouse models have contributed to our understanding of arrhythmogenesis in these conditions [1], [2], [3], [4], [5], [6], [7], [8], [9]. Primary inherited arrhythmic syndromes are now recognized to be more complex than abnormality in a particular ion channel, involving alterations in gene expression and structural remodelling [10], [11]. Conversely, in cardiomyopathies and heart failure, mutations in ion channels and proteins have been identified as underlying causes, and electrophysiological remodelling are recognized pathological features. Transgenic techniques causing mutagenesis in mice are extremely powerful in dissecting the relative contributions of different genes play in producing disease phenotypes. The importance of translational research cannot be overstated. As previously suggested, a better classification is myocardial disease involving abnormalities in one of the following cellular components: cytoskeleton, desmosome, sarcomere and ion channels [29]. Overlap syndromes between different cardiomyopathies are now increasing recognized but are nevertheless uncommon in humans, and indeed rarer than individual cardiomyopathy occurring in isolation. Thus, overlapping features between DCM and HCM, ARVD and HCM, ARVD and DCM, and post-partum cardiomyopathy (PPCM) and ARVD [176] have all been described. Mouse models can serve as useful systems in which to explore how protein defects contribute to arrhythmias and direct future therapy.

## Conflict of interest

The authors declare no conflict of interest.
